# Synergetic effect of TiC particles on cavitation erosion resistance of AZ31-TiC surface composites

**DOI:** 10.1016/j.heliyon.2025.e42602

**Published:** 2025-02-10

**Authors:** T Satish Kumar, S. Shalini, Robert Čep, Kanak Kalita

**Affiliations:** a Department of Mechanical Engineering, Amrita School of Engineering, Amrita Vishwa Vidyapeetham, Coimbatore 641112, India; b Department of Physics, PSG Polytechnic College, Coimbatore, Tamil Nadu, India; c Department of Machining, Assembly and Engineering Metrology, Faculty of Mechanical Engineering, VSB-Technical University of Ostrava, 70800 Ostrava, Czech Republic; d Department of Mechanical Engineering, Vel Tech Rangarajan Dr. Sagunthala R&D Institute of Science and Technology, Avadi 600 062, India; e Jadara Research Center, Jadara University, Irbid, 21110, Jordan

## Abstract

AZ31/TiC surface composites were produced using Friction Stir Processing (FSP), with varying amounts of TiC particles. The microstructure and hardness measurements, as well as the evaluation of erosion wear resistance, were carried out on AZ31/TiC composites. X-ray diffraction tests were carried out to identify the phase composition. The presence of α-Mg and TiC phases was observed in all composites and no chemical interactions between the AZ31 matrix and TiC were observed at the interface. The AZ31 alloy is shown to have a hardness of 62 The AZ31 alloy reinforced with 15 vol% of TiC particles showed the highest resistance to cavitation with volume loss of 44 mm^3^, while the AZ31 alloy showed the lowest resistance with volume loss of 142 mm^3^ for 15 min exposure time. HV ± 2 HV, whereas the AZ31/15 vol% of TiC composites is found to exhibit the highest hardness of 116 HV ± 5 HV.

## 1 Introduction

Due to its lightweight nature, magnesium has good mechanical properties and can be used in a wide range of applications, both structural and non-structural [[1], [2], [3], [4], [5], [6]]. With its capacity to diminish air, water, land, and electromagnetic contamination, this metal has the potential to be a sustainable alternative to aluminium. It is also 12 times more abundant than aluminium, offering a possible solution on a global scale. Over the past two decades, there have been significant research efforts to incorporate magnesium into prominent applications. This has been achieved gradually, with notable contributions from the automobile, aircraft, electronics and biomedical industries. Due to the significant prospects of magnesium, numerous magnesium alloys have been researched and made available for industrial applications [7]. Nevertheless, an area with significant promise that has not been thoroughly explored is the realm of magnesium composite technology [8,9].

Bagheri et al. [10] demonstrates the effectiveness of friction stir vibration processing (FSVP) as a modified approach to friction stir processing (FSP) for enhancing the properties of AZ91 magnesium alloy surface composites. The application of vibrations normal to the processing direction resulted in a more homogeneous distribution of SiC particles and finer grain structures (26.43 ± 2.00 μm) compared to FSP (39.43 ± 2.00 μm). FSVP also improved mechanical properties, including ultimate tensile strength (361.82 MPa), elongation (16.88 %), and formability index (6107.52 MPa%), compared to FSP. The increased dynamic recrystallization due to workpiece vibration was identified as the key factor contributing to these improvements, with higher vibration frequencies amplifying the effects. Junhao Liang et al. [11] used a novel method combining friction stir processing (FSP) and ultrasonic-assisted extrusion was developed to fabricate CNTs-reinforced AZ91D magnesium nanocomposites. This approach achieved excellent dispersion of CNTs, resulting in significant strength enhancement due to dislocation strengthening and stress transfer mechanisms, without sacrificing ductility. The composites exhibited reduced Mg_17_Al_12_ phase content with increasing CNT concentration and strong interfacial bonding, confirmed by the formation of Al_2_MgC_2_ at the CNTs-matrix interface.

Jonda et al. [12] examined the influence of mechanical characteristics and microstructural attributes on the sliding wear and cavitation erosion phenomena of cermet coatings applied through high-velocity oxy-fuel (HVOF) spraying onto an AZ31 magnesium substrate. The results of cavitation testing demonstrated that the WC-Co-Cr cermet exhibited a significantly greater resistance to cavitation erosion compared to WCCr_3_C_2_-Ni and WC-Co coatings and exhibited markedly superior resistance relative to the AZ31 substrate, as evidenced by the respective volumetric material losses of 3.74 mm³, 6.99 mm³, 10.30 mm³ and 108.82 mm³. The erosion mechanism of the WC-Co-Cr coating was characterized by a uniform removal of material, which contributes to a reduced erosion rate. The CoCr binder effectively mitigated the occurrence of severe surface pitting and the detachment of the WC-Co-Cr cermet material in substantial fragments, a phenomenon that was observed in the WC-Co and WCCr_3_C_2_-Ni coatings. The application of cermet coatings provided an efficient protective barrier for the magnesium substrate, which inherently exhibits low resistance to cavitation erosion and sliding wear in the absence of a coating. Mg and Ti possess non-toxicity, exceptional biocompatibility and mechanical qualities [13]. As a result, Mg composites have a wide range of prospective applications in various technical fields, such as biomedical and transportation industries. Automobile and aircraft applications pose a possibility of dynamic stress and therefore, it is crucial for the materials used in these applications to endure such loading circumstances. Given the excellent quasi-static mechanical capabilities exhibited by Mg composites, it is necessary to study the impact resistance of these composites to make them appropriate for the transportation sector.

Cavitation erosion tests enable the assessment of a material's ability to withstand repeated high-speed impacts. Cavitation erosion is the process by which a material's surface degrades due to repeated hits from cavitation impulses. These impulses are micro-jets that occur when cavitation bubbles implode. Cavitation bubbles form in a liquid when the pressure drops rapidly below the vapour pressure in a specific area. When the pressure of the liquid surrounding the bubble becomes greater than the pressure inside the bubble, the bubble collapses due to cavitation. This collapse generates a shock wave and a micro-jet. The implosion of cavitation bubbles upon gets in touch with the rendering material causing the most extreme loading. As the distance between the collapsing cavitation bubble and the material surface increases, the velocity and pressure of the impact diminishes as the velocity of striking micro-jets ranges from a few m/s to several 100 m/s. The micro-jets produce impact stress varying from a few KPa to several GPa [14]. The high intensity of cavitation micro-jets and their brief duration of impact result in the term “cavitation pulses.” As the fluid flow velocity increases, the number and strength of cavitation bubbles in the flowing cavitation also increases. Prior studies have demonstrated that the resistance of a material to degradation caused by cavitation erosion is strongly correlated with its durability characteristics, specifically its fatigue strength and hardness [15]. The resistance of cavitation erosion can be affected by material properties such as impact energy as determined by the mechanism of surface degradation.

Magnesium-titanium composites demonstrate a remarkable compressive strength of up to 175 MPa. Moreover, as the titanium content rises, the compressive strength also increases. Shivali Singla et al. [16] developed surface composites of WE43 magnesium alloy enhanced with titanium carbide (TiC) via friction stir processing (FSP). The grain size of the WE43 Mg alloy has significantly decreased from 22.42 to 6.6 μm. Additionally, the treated composite with square-shaped tool-pin geometry achieved a maximum micro-hardness level of 180 HV 0.3. Furthermore, the corrosion rate of the composite is determined to be 45 % lower than that of the WE43 Mg alloy. Prem Sagaret al. [17] produced magnesium alloy with titanium carbide particles by employing the FSP technique. A substantial increase of approximately 2.68 times in microhardness values, accompanied by a corresponding reduction in grain size of up to 14.85 times, was achieved. The ultimate tensile and compressive strengths exhibit a remarkable increase in values, reaching up to 2.16 and 1.6 times respectively, in comparison to the base metal. Regarding wear morphology, the base magnesium alloy showed abrasive and adhesive wear, but the composite displayed delamination, particle pull-out and localized plastic deformation, along with improved wear resistance. It was observed that the specimens' hardness and resistance to cavitation erosion were significantly enhanced using FSP. This improvement is attributed to the microstructure refinement and the uniform distribution of hard reinforcements in the Mg matrix. From the literature survey, it was also observed that very few studies are available on the cavitation erosion behavior of AZ31/TiC surface composites. Hence, the objective of this investigation is to assess the influence of TiC concentration on the long-term resistance of AZ31/TiC surface composites against cavitation erosion.

## 2 Methodology

### 2.1 Materials used

A hot-rolled plate (160 mm × 80 mm × 6 mm) made of AZ31 magnesium alloy consisting of 4 % Al, 1.2 % Zn, 0.5 % Mn and the Mg was used as the matrix material. TiC particles with 10 μm size were used as reinforcement.

### 2.2 Methodology for processing AZ31/TiC composites

Three different composites with 5 vol%, 10 vol% and 15 vol%, TiC particles were synthesized using an indigenous developed FSP technique. These FSP steps included groove cutting, filling the grooves with the TiC reinforcements and applying shallow plastic deformation without the use of a pin to encapsulate the powder. Subsequently, FSP of the prepared specimens were carried out using H13 steel tool. The tool dimensions were a tilt angle of 2°, 18 mm for the shoulder diameter, 6 mm for the pin diameter, a plunging depth of 0.8 mm and 6 mm for the pin length. The parameters for FSP were carried out with a traverse speed of 100 mm/min, a 4 kN axial force and a rotational speed of 900 rpm. Since all composites were manufactured using identical conditions, the current study disregards the impact of processing characteristics.

### 2.3 Microstructural characterization

The microstructure of the AZ31/TiC composites was analyzed using Nikon make optical microscope (MA-100) and a Gemini 300 make Scanning Electron Microscope (SEM) equipped with EDS. The composite specimens were polished and etched by means of an etchant solution containing 20 ml of distilled water, 1 ml of nitric acid, 10 ml of acetic acid and 60 ml of ethylene glycol. The grain size was determined using the line intercept method [8]. Furthermore, X-Ray diffraction analysis was conducted using a Shimadzu make X-Ray diffractometer −6000 using (Cu Ka; λ = 1.54056 A^o^) at 2°/min scan speed.

### 2.4 Mechanical characterization

AZ31/TiC composites were evaluated for their hardness. This examination utilized a Mitutoyo make HM-200 Micro Hardness testing machine with a load of 100 gf and a dwell time of 15 s. The hardness measurements were conducted in accordance with the ASTM standard E384-11E1. Hardness was measured prior to the erosion tests and after a 5-min testing period. Each sample was subjected to 10 sets of measurements. The hardness of AZ31/TiC composite was determined by calculating the average of the measurement hardness values.

### 2.5 Erosion test

**Fig. 1:**
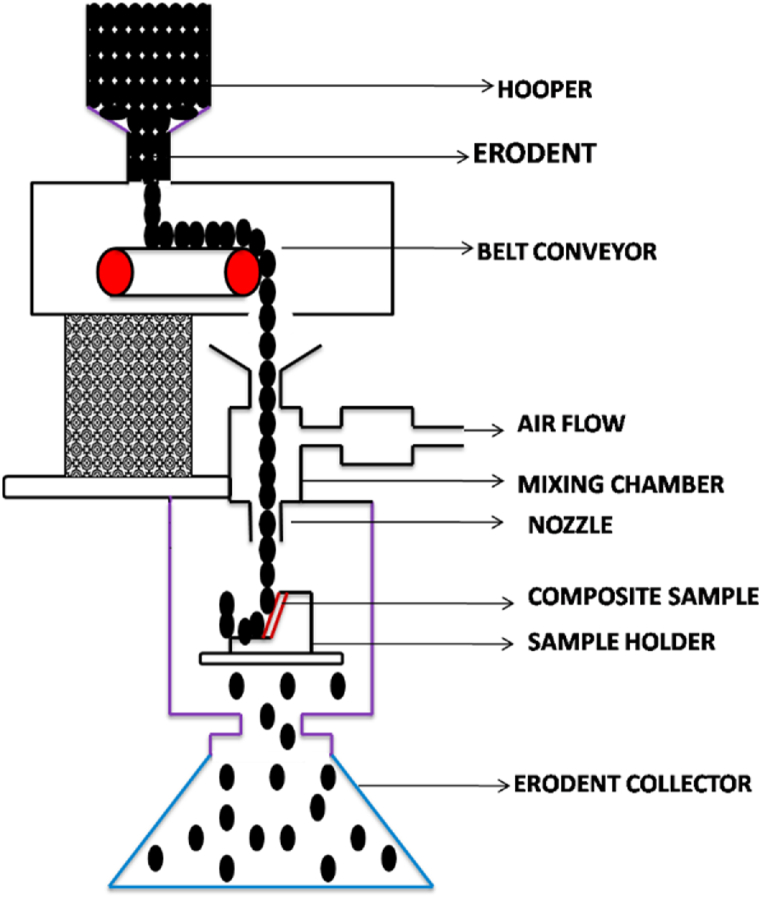
Schematic diagram of erosion tester.

Cavitation erosion resistance experiments were conducted utilizing a spinning disc system. This configuration produces an exceptionally high level of cavitation aggressiveness. The rotational velocity was 2900 rpm. The revolving disc, measuring 314 mm in diameter contains cavitation Inductors. These inductors are cylindrical steel bolts measuring 12 mm in diameter and 8 mm in height. Furthermore, the disc also contains samples of the material being investigated, having a diameter of 30 mm (Fig. 1). The arrangement of the rotating disc enables the simultaneous assessment of four samples. The experiment included three 5-min exposures, leading to cumulative test duration of 15 min. Prior to conducting the tests and after each specified time, the samples underwent a process of cleansing, desiccation, and measurement using an analytical scale with a precision of 0.1 mg. Since the density of each composite is affected by the quantity of Ti particles, the cavitation curves were displayed as a plot of the reduction in volume vs the duration of exposure. Surface damage was detected following the cavitation erosion experiments.

## 3 Results and discussion

### 3.1 Microstructure and mechanical characterization

**Fig. 2:**
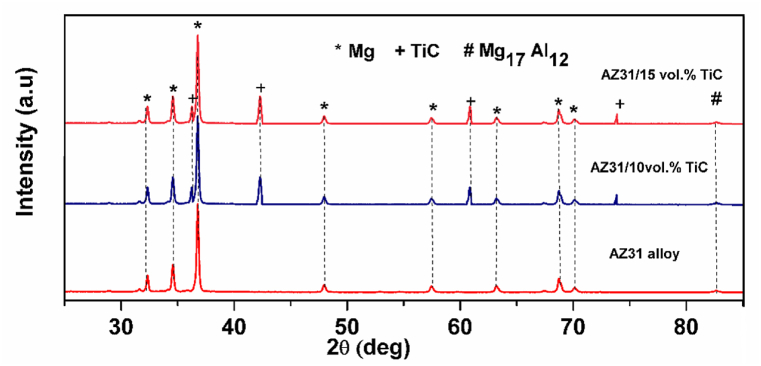
XRD pattern for AZ31 alloy and AZ31/TiC composites.

Fig. 2 presents the XRD patterns corresponding to the AZ31 alloy and the AZ31/TiC FSP composites. The distinctive peaks related to the magnesium matrix, Mg_17_Al_12_ and the reinforcing compound TiC are clearly discernible in Fig. 2. Notably, there is an absence of significant evidence indicating the presence of any detrimental phases within the composite samples analyzed. The immiscibility of magnesium and TiC suggests that these materials do not easily dissolve in one another, regardless of the thermal conditions.

**Fig. 3:**
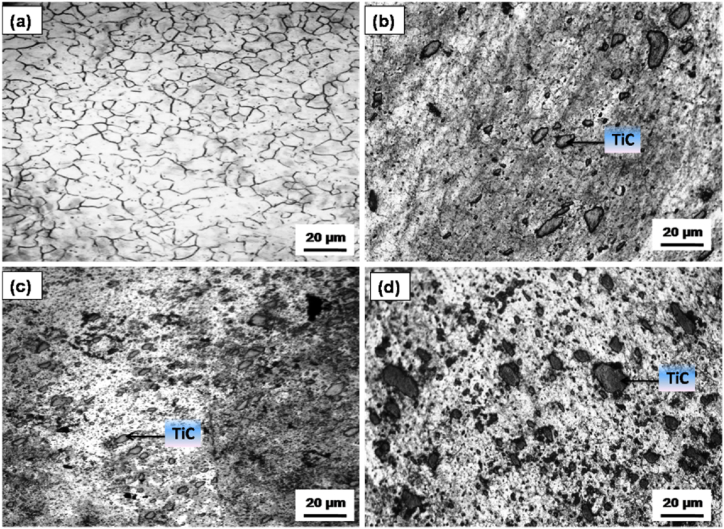
Micrographs of (a) AZ31 alloy, (b) AZ31/5 vol% TiC, (c) AZ31/10 vol% TiC and (d) AZ31/15 vol% TiC FSP composites.

**Fig. 4:**
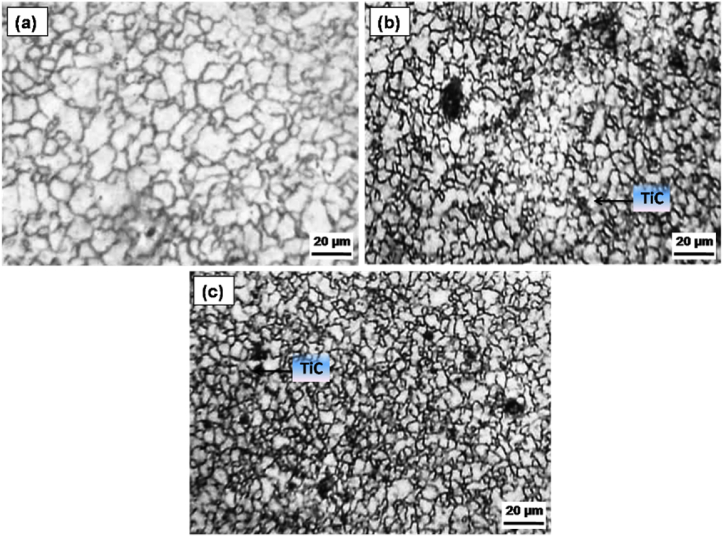
displays the grain size of the (a) AZ31/5 vol% (c) AZ31/10 vol% and (d) AZ31/15 vol% TiC FSP composites.

**Fig. 5:**
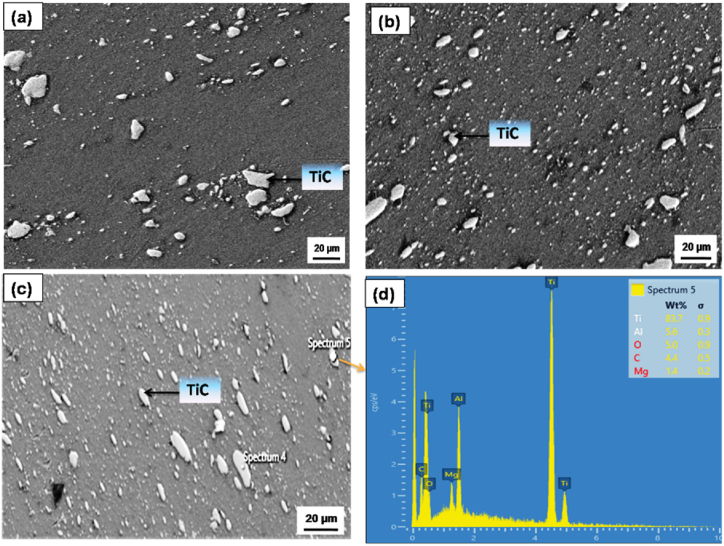
SEM images of (a) AZ31/5 vol% (b) AZ31/10 vol%, (c) AZ31/15 vol% TiC FSP composites and (d) EDS spectrum of the AZ31/15 vol% TiC composite**.**

Fig. 3a–d illustrates the microstructural characteristics of the AZ31 alloy and composites with 5, 10 and 15 vol% TiC respectively. The microstructure reveals a homogeneous distribution of TiC particulates within the AZ31 matrix, devoid of any aggregation. The uniformity in the distribution of reinforcement particulates is anticipated to enhance the mechanical properties of the composite significantly. The XRD peaks corroborate the existence of magnesium (Mg) along with TiC as constituent phases in the composite. This observation substantiates the assertion that no interaction transpires between the reinforcement particulates and the matrix. Fig. 4a–c shows the grain size of the AZ31/5, 10 and 15 vol% TiC composites respectively. The as-received base magnesium alloy (AZ31B) is characterized by coarse grains, exhibiting a grain size approximately in the vicinity of 60 μm (Fig. 3a). The grains were observed to undergo significant refinement due to the dynamic recrystallization phenomena that transpired throughout the FSP. This grain refinement is projected to culminate in the generation of fine equiaxed grains with a grain size ranging from about 6 to 8 μm in 15 vol% TiC composites as demonstrated in Fig. 4c. The incorporation of small TiC particles at the grain boundaries of the matrix, together with dynamic recrystallization (DRX) induced by the tool, enhances the process of grain refinement in the composite material [[18], [19], [20]]. Fig. 5 shows the SEM images of (a) AZ31/5 vol% (b) AZ31/10 vol%, (c) AZ31/15 vol% TiC FSP composites and (d) EDS spectrum of the AZ31/15 vol% TiC composite**.** The particles are surrounded by a continuous interface, which is free from any interruptions and no foreign particles are observed at this contact. AZ31 alloy is effectively reinforced with particles on all sides, exhibiting a pore-free structure. Furthermore, the TiC reinforcement particles do not chemically react with the AZ31 alloy confirming no secondary phase is observed. EDS spectrum of the AZ31/15 vol% TiC composite confirms the presence of added TiC particles in the AZ31 matrix.

**Fig. 6:**
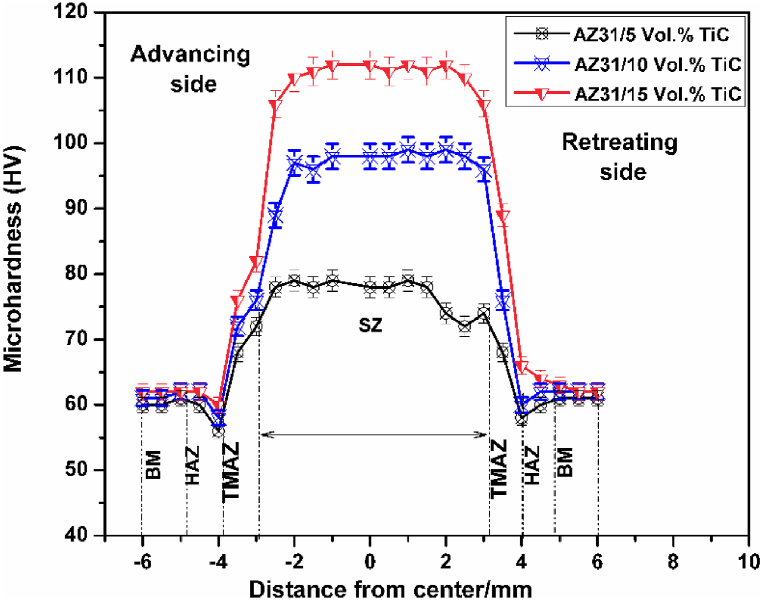
Hardness graph for AZ31/TiC FSP composites.

Hardness graph for AZ31 alloy and AZ31/TiC FSP composites is depicted in Fig. 6. The AZ31 alloy is shown to have a hardness of 62 HV ± 2 HV, whereas the matrix of AZ31/TiC composites is found to exhibit hardness ranging from 72 ± 3 HV to 116 HV ± 5 HV. Based on the composite structure (Fig. 5), the variation in hardness is likely to be caused by the presence of reinforcing particles that may have been present beneath the indenter during the hardness tests. In addition, the TiC particles reduce the size of the grains in the AZ31 matrix. The AZ31/TiC composites exhibit enhanced hardness due to their smaller grain size. Consequently, the mechanical characteristics of the composites are enhanced. As the volume percentage of TiC particles increases, the influence of the previously stated mechanisms also increases, leading to a decrease in the average distance between particles thereby enhancing the resistance to the movement of dislocations [21,22]. Consequently, as the volume percentage of TiC particles increases, the mechanical properties of the composites also increase. Intense plastic deformation of FSP/FSW leads to a rise in the density of dislocations, which subsequently enhances the microhardness [23,24].

### 3.2 Cavitation erosion properties of AZ31 alloy and its composites

**Fig. 7:**
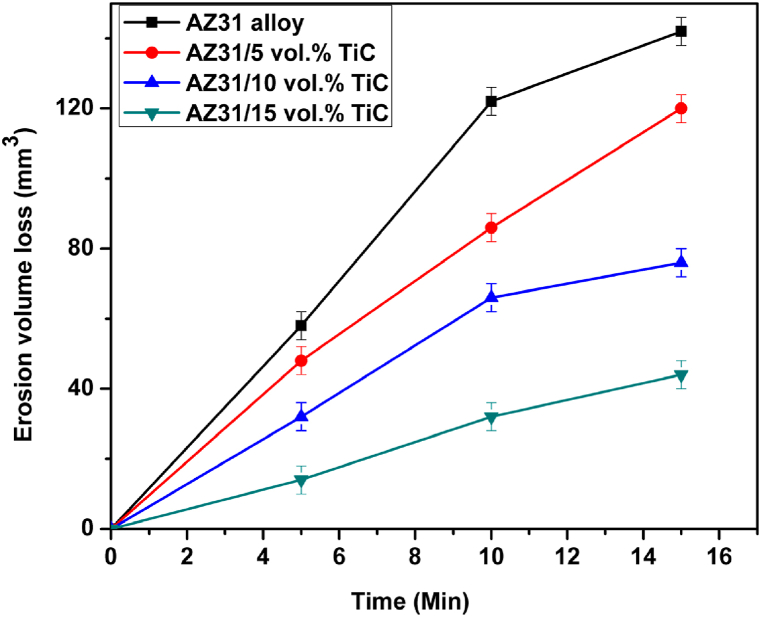
Cavitation erosion plot of AZ31 alloy and its FSP composites.

Fig. 7 displays the cavitation curves, which represent the relationship between volume loss and exposure time. The AZ31 alloy was found to show a maximum volume loss, followed by AZ31/5 vol% TiC composite, the AZ31/10 vol% TiC composite and finally the AZ31/15 vol% TiC composite. Thus, the composites ranking in terms of their resistance to cavitation erosion constantly corresponds to the increasing quantity of TiC particles. The ability to cope with cavitation erosion is determined by the mechanical and durability qualities of materials. The potential for strain-hardening is a crucial material feature that directly affects its resistance to cavitation erosion. A measurement was carried out to evaluate the surface hardness of the AZ31 alloy and the composite materials following a 5-min testing session. The data presented in Table 2 shows that the surface hardness of AZ31/15 vol% TiC composites increased from 112 HV ± 4 HV to 128 HV ± 5 HV. The observed rise in the variations of hardness measurements, in comparison to the first data, can likely be attributed to the random impact locations of the cavitation pulses [21].

**Table 2:** Hardness value after 5 min of cavitation erosion testing.

Alloy/Composite	Hardness (HV)
AZ31 Alloy	72 ± 3
AZ31/5 vol% TiC	89 ± 3
AZ31/10 vol% TiC	114 ± 4
AZ31/15 vol% TiC	128 ± 5

Additionally, the presence of hard TiC particles may also contribute to this phenomenon. The conducted research has revealed a noteworthy level of cavitation intensity, as the occurrence of strain-hardening in cavitation erosion tests is strongly linked to cavitation intensity [9]. In addition to strain-hardening, the resistance to cavitation erosion is also influenced by parameters such as yield stress and fracture strain. The properties of AZ31/TiC composites are influenced by the amount of TiC particles. The studies conducted by Esen et al. [25] showed that the addition of titanium (Ti) to magnesium (Mg) composites leads to an increase in both the yield stress and ultimate compression strength.

**Fig. 8:**
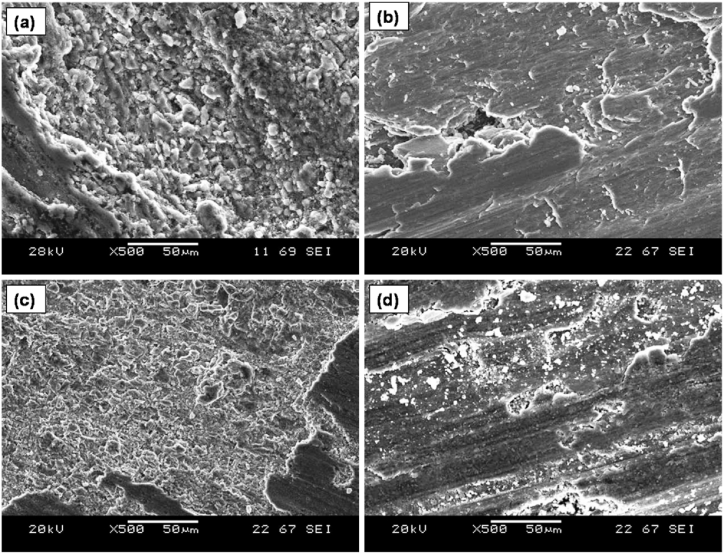
SEM images of damages formed during cavitation erosion test (a) AZ31 alloy, (b) AZ31/5 vol% TiC, (c) AZ31/10 vol% TiC (removal of hard TiC particles) and (d) AZ31/15 vol% TiC development of cracks at Ti particle of FSP composites.

To improve the understanding of the AZ31/TiC composites resistance to cavitation erosion and the mechanism of degradation, microscopic examinations were carried out to analyze the damage. Fig. 8 depicts the whole regions of wear on the AZ31 alloy and its studied composites. The surface of the AZ31 alloy Fig. 8a exhibited the most severe erosion damage. The damages exhibited were both equivalent and profound, which aligns with the cavitation erosion curves as seen in Fig. 5. The AZ31/10 vol% TiC (Fig. 8c) and AZ31/15 vol% TiC (Fig. 8 d) composites exhibited less cavitation erosion compared to the AZ31 alloy. A noticeable cavity can be observed in the area impacted by cavitation in the AZ31 alloy (Fig. 8a). In the case of the AZ31/15 vol% TiC composite (shown in Fig. 8d), the extent of damage was considerably less deep. Therefore, the observations of all surfaces that were damaged were in agreement with the loss of volume, as shown in Fig. 7.

The surface of both AZ31 and AZ31/5 vol% TiC composites experienced plastic deformation, as seen in Fig. 8a. The occurrence of ductile surface deformation is highly unexpected as magnesium is recognized for its limited ductility resulting from its hexagonal close-packed structure (HCP) structure. Given that elongation is seen as a quantification of ductility, it is necessary to conduct a more in-depth analysis of this phenomenon. According to the references by Hassan et al. [26] the elongation of pure magnesium under quasi-static strain is approximately 7.7–8%. The inclusion of TiC particles in AZ31 Mg alloy and the resulting production of AZ31/TiC composites have been found to lead in reduction in ductility compared to static tests. Due to high-speed deformation, the ductility of the material is reduced, resulting in decreased elongation of AZ31 and composites under cavitation.

The low ductility of AZ31 alloy under quasi-static stress is attributed to its crystalline structure, which is characterized by a limited number of slip systems that facilitate deformation and high stacking fault energy. Thus, it is extremely difficult to cause a slip, especially in slip systems other than the basal plane, at normal temperatures. High stacking fault energy of Magnesium also enables deformation by twinning. The distortion observed in AZ31 and AZ31/TiC composites can be attributed to twinning. Another significant outcome of cavitation erosion was the development of considerable tunnels, as illustrated in Fig. 6b. When a micro-jet with high velocity impacts the surface of AZ31 and AZ31/TiC composites, it produces shear and compressive waves. According to Chichili et al. [27], an increase in strain rate from 1.16 × 10^−5^ s^−1^ to 6 × 10^3^ s^−1^ resulted in an augmentation in compressive flow, shear stress and strain hardening. Adiabatic thermal softening is another result of high velocity impact. This softening, along with intense shear stress, promotes the development of micro-tunnels [28]. The dimensions of the cavitation tunnel that was measured are greater than 200 μm in size/diameter. Cavitation tunnels are linked to the development of cavitation erosion, which is caused by the intense speed of micro-jets and the rapid deformation of the AZ31 matrix resulting from the impact of these micro-jets. The dimensions of the tunnels are determined by the characteristics of the AZ31/TiC composite, which are associated with its HCP structure and the relatively low hardness of the AZ31 matrix.

The subsequent damages that were observed comprised of craters, which were created because of the extraction of TiC particles (Fig. 6 c). The existence of these craters, which resemble pores like shape, increases the pace of erosion by causing variation in the flow conditions. The formation of craters serves as obstacles for the movement of liquid, resulting in the generation of more cavitation bubbles. Their collapses contribute to the ongoing enlargement of craters. If a pore develops beneath the crater, it undergoes a metamorphosis into a deep tunnel, leading to an increased erosion rate. Craters also function as areas of stress concentration and serve as starting points for cracks in AZ31. Hence, the abundance of several expansive craters adversely affects the capacity to endure cavitation. The topmost layer of the AZ31 matrix underwent plastic deformation and strain-hardening due to cavitation erosion. Plastic deformation and surface hardening provided evidence of the existence of deformation twins in the AZ31 Mg matrix. TiC particles are used as reinforcements in the AZ31/TiC composite because of its superior shear and elastic modulus, in addition to increased hardness in comparison to the AZ31 Mg alloy.

As a result, TiC particles can withstand the erosion cavitation and significantly reduces the erosion wear of composite as shown in Fig. 8d. The presence of TiC particles in the composites will not allow the crack to propagate easily in the matrix and further reduces the volume loss of the matrix. Erosion tested surface of the AZ31 alloy shows severe and deep cavitation tunnels as observed in Fig. 8a, where the composite sample shows shallow cavitation with less material loss. The degradation of the AZ31/TiC composite is attributed to the development of cracks at the interface among the TiC particles and the AZ31 matrix, leading to the detachment of the TiC particles. Upon closer examination of composite fracture exhibiting quasi cleavage was discovered beneath the outer layer.

## 4 Conclusions

The investigations on the resistance to cavitation erosion of AZ31 alloy and AZ31/TiC composites demonstrate that the microstructure of the AZ31/TiC (5, 10, and 15 vol%) composites exhibited a consistent distribution of TiC particles inside the magnesium matrix, resulting in a refinement of the matrix. The microhardness values revealed that AZ31/15 vol% TiC consists of a highest hardness of 116 HV compared to AZ31 alloy hardness of 62 HV. The composite material AZ31/15 vol% TiC demonstrated the least amount of cavitation erosion, while the AZ31 alloy had the highest level of cavitation erosion. The AZ31 alloy reinforced with 15 vol% of TiC particles showed the highest resistance to cavitation with volume loss of 44 mm^3^, while the AZ31 alloy showed the lowest resistance with volume loss of 142 mm^3^ for 15 min exposure time. Erosion tested surface of the AZ31 alloy shows severe and deep cavitation tunnels, where the composite sample shows shallow cavitation with less material loss.

In future work, investigating the long-term stability of AZ31/TiC composites under diverse environmental conditions, such as corrosion and elevated temperature exposure, would provide valuable insights into their performance and durability for practical applications.

## Funding

This work was funded by the European Union under the REFRESH-Research Excellence For REgion Sustainability and High-tech Industries project number CZ.10.03.01/00/22_003/0000048 via the Operational Programme Just Transition.

## CRediT authorship contribution statement

**T Satish Kumar:** Writing – original draft, Validation, Resources, Methodology, Investigation, Formal analysis, Data curation, Conceptualization. **S. Shalini:** Writing – original draft, Validation, Methodology, Investigation, Formal analysis, Data curation, Conceptualization. **Robert Čep:** Writing – review & editing, Methodology, Funding acquisition, Conceptualization. **Kanak Kalita:** Writing – review & editing, Methodology, Funding acquisition, Conceptualization.

## Declaration of competing interest

The authors declare that they have no known competing financial interests or personal relationships that could have appeared to influence the work reported in this paper.
